# Trefoil factor 3 can stimulate Th17 cell response in the development of type 2 diabetes mellitus

**DOI:** 10.1038/s41598-024-60426-7

**Published:** 2024-05-06

**Authors:** Ziyang Lin, Jinyuan Zhang, Tingting Duan, Junzheng Yang, Yiqi Yang

**Affiliations:** 1 Key Laboratory of Glucolipid Metabolic Diseases of the Ministry of Education, Guangdong Metabolic Diseases Research Center of Integrated Chinese and Western Medicine, The Institute of Chinese Medicinal Sciences, Science and Technology Building, Guangzhou Higher Education Mega Centre, Guangdong Pharmaceutical University, 280 Wai Huan Dong Road, Guangzhou, 510006 People’s Republic of China; 2 Guangdong Nephrotic Drug Engineering Technology Research Center, Institute of Consun Co. for Chinese Medicine in Kidney Diseases, Guangdong Consun Pharmaceutical Group, Guangzhou, People’s Republic of China

**Keywords:** Helper T Lymphocyte 17, Inflammation, Trefoil factor 3, Type 2 diabetes mellitus, Immunology, Endocrinology

## Abstract

This study aims to evaluate the role of trefoil factor 3 (TFF3) peptides in type 2 diabetes mellitus (T2DM) from an inflammatory perspective. The focus was on exploring how TFF3 affects the function of T cells. TFF3 overexpression model was constructed using lentivirus in Jurkat cell lines. We evaluated the impact of TFF3 on the proliferation, apoptosis, and IL-17A levels of Jurkat cells cultured in high glucose. The T2DM model was induced in TFF3 knockout (KO) mice through streptozotocin combined with high-fat diet. The measurements included glucose tolerance, insulin tolerance, inflammation markers, Th17 cell proportion, and pancreatic pathological changes. The T2DM modeling led to splenomegaly in mice, and increased expression of TFF3 in their spleens. Overexpression of TFF3 increased the proportion of IL-17^+^ T cells and the levels of Th17-related cytokines in Jurkat cells. There was no difference in body weight and blood glucose levels between wild-type and TFF3 KO mice. However, T2DM mice lacking the TFF3 gene showed improved glucose utilization, ameliorated pancreatic pathology, decreased inflammation levels, and reduced Th17 cell ratio. TFF3 may be involved in the chronic inflammatory immune response in T2DM. Its mechanism may be related to the regulation of the RORγt/IL-17 signaling pathway and its impact on T cell proliferation and apoptosis.

## Introduction

According to the International Diabetes Federation, diabetes affected 425 million people globally in 2017, and this number is projected to rise to 783 million by 2045 (https://diabetesatlas.org/). Diabetes mellitus, known for its high incidence, involves a complex pathogenesis and potential for multiple complications. Research increasingly portrays diabetes as a chronic inflammatory disease, aggravated by inflammatory factors produced due to immune cell infiltration and polarization^1–3^. Notably, the activation of T helper cell 17 (Th17) is a pivotal mechanism in chronic inflammation. The interleukin (IL)-17 specifically secreted by Th17 cells can harm pancreatic islet cells, worsening insulin resistance and initiating inflammatory pathways in renal parenchymal cells, ultimately causing diabetic kidney injury^4,5^. Therefore, regulating Th17 activity has become a crucial strategy for alleviating chronic inflammation in diabetes. However, the upstream factors that activate Th17 cells remain poorly understood. Identifying potential factors or targets to promote the differentiation of lymphocytes into inflammatory subtypes, thus restoring immune balance in a timely manner, holds great significance in delaying the progression of diabetes and its associated complications.

Recent studies have shown that trefoil factor family protein 3 (TFF3) is significantly elevated in patients with diabetes^6–9^, which is a small molecular peptide with potential immunomodulatory activity^10,11^. For instance, TFF3 knockout (KO) mice, in contrast to their wild-type (WT) counterparts, exhibited a milder inflammatory response in an ileitis model. This was characterized by reduced expression of toll-like receptor 4, interferon-γ, IL-12, IL-1β, tumor necrosis factor (TNF)-α, and a decreased number of CD4-positive lymphocytes in the ileum^12,13^. Mice on a high-salt diet also displayed a dampened inflammatory response due to TFF3 gene depletion, resulting in fewer CD25^+^CD4^+^T cells in lymph nodes^14^. Some researchers have proposed that TFF3 may directly regulate immune responses as an intrinsic secretory product of immune cells^12^. This hypothesis is based on the report that TFF3 and TFF2 synthesis in lymphoid tissues, such as the spleen, thymus, lymph nodes, or bone marrow, which can stimulate monocyte migration^15^. Moreover, TFF3 is produced in vitro by activated microglia, a major immune cell found in the central nervous system^16^. Regarding TFF2, another member of the TFF family, there is compelling evidence of its direct role in regulating immune responses^17^.

For a considerable duration, TFF3 research has predominantly revolved around cancer, with limited attention given to its relevance in the realm of metabolic diseases. Existing studies have primarily concentrated on assessing TFF3 changes in diabetic patients or examining the potential of TFF3 as a diagnostic biomarker for diabetes. The significance of pathologically elevated TFF3 and its potential involvement in Th17 cell differentiation have remained largely unexplored. Therefore, our study intend to investigate the role of TFF3 in high glucose-cultured T lymphocytes, as well as in a mouse model of type 2 diabetes mellitus (T2DM). We have elucidated the molecular mechanisms underlying TFF3’s role in chronic inflammation. These findings hold the potential to unveil new therapeutic targets for the treatment of metabolic inflammatory disorders.

## Materials and methods

### Reagents and chemicals

Streptozotocin (STZ) was purchased from Sigma-Aldrich (Catalog #S0130, St.Louis, MO, USA). The high-fat diet (HFD, 60% calories from fat) was purchased from Dyets Inc (Catalog #HF60; Betheleham, PA, USA). ELISA kits for IL-17A, TNF-α, IL-6, and IL-23 were purchased from MEIMIAN (Jiangsu, China). Antibodies against β-actin (#3700S), GAPDH (#5174), anti-rabbit IgG HRP-linked (#7074) and anti-mouse IgG HRP-linked (#7076) antibodies were purchased from Cell Signaling Technology Inc. (Beverly, MA, USA). Antibodies against RORγ (#ab113434), IL-17A(#ab193955), and TFF3(#ab272927) were obtained from Abcam (Cambridge, USA). Antibodies against TFF3 Alexa Fluor^®^ 488(#sc-398651 AF488) were purchased from Santa Cruz (Dallas, USA). The bicinchoninic acid (BCA) protein assay kit was purchased from CoWin Biosciences (CW0014, Shanghai, China).

### Plasmid construction and cell transfection

The Jurkat T cell line is provided by Hanheng Biotechnology (Shanghai, China), which also provides stable TFF3 overexpression cell services. Jurkat cell were cultured in RPMI 1640 complete medium (Thermo Fisher Scientific, Inc.). Lentivirus packaging was performed using a triple-plasmid system: a vector plasmid carrying the TFF3 gene, psPAX2, and pMD2G vector plasmid. Co-transfection into 293T cells was facilitated by Lipofiter™ transfection reagent. Virus supernatant, collected, concentrated, purified, and assessed for quality, was used to infect Jurkat T cells with lentiviruses—control virus HBLV-ZsGreen-PURO and target virus HBLV-h-TFF3-ZsGreen-PURO. Stable TFF3-expressing strains were obtained by selection with puromycin and observed under immunofluorescence microscope.

### Quantitative real-time PCR (qPCR)

Gene overexpression was confirmed through qPCR. Total RNA was extracted from the cells using TRIZOL reagent. GAPDH was used as the reference gene. The primer sequences are as follows: TFF3, 5-GGGGCTGCTGCTTTGACT-3′ (forward) and 5-AAGGTGCATTCTGCTTCCTG-3′ (reverse); GAPDH, 5-TCAAGGCTGAGAACGGGAAG-3′ (forward) and 5-TCGCCCCACTTGATTTTGGA-3′ (reverse).

### Generation and genotyping of TFF3^KO^ mouse

Male TFF3^KO^ mice (Strain NO. T028324) were purchased from GemPharmatech (Nanjing, China). Using the CRISPR/Cas9 genome-editing technology, male C57BL/6J mice were introduced a 3834-bp deletion within the exon of TFF3. Male WT mice were purchased from Vital River Laboratory Animal Technology Co., Ltd, Beijing, China (Animal Certificate No. 110011211102655721). KO mice were crossed with WT mice to generate syngeneic offspring with the desired genotypes. All offspring were born normally at the expected Mendelian frequency. Genotypes were identified by a routine PCR protocol for tissues with the following genotyping ear punch primer pairs: forward 5′-CCAAATCTCTAAGGATACAAACCACAGC-3′ and reverse: 5′-CTGGGTAACGTGTTCACCTCACATG-3′ which yield 4184-bp and 350-bp amplicons, respectively, for the WT and KO alleles. All experimental protocols were approved by the Experimental Animal Ethics Committee of Guangdong Pharmaceutical University. All methods were carried out in accordance with relevant guidelines and regulations. All methods are also reported in accordance with ARRIVE guidelines.

### T2DM model

Mice were divided into two groups: Control (standard diet) and T2DM (high-fat diet). After four weeks on the high-fat diet, the T2DM group received intraperitoneal injections of STZ (40 mg/kg/d) for five consecutive days^5^, while the Control group received an equivalent citrate solution. Fasting blood glucose levels were measured six hours after the final injection, and levels exceeding 11.1 mmol/L confirmed T2DM establishment. Samples were collected after 12 weeks of high-fat diet feeding^5^. All experimental protocols were approved by the Experimental Animal Ethics Committee of Guangdong Pharmaceutical University. All methods were carried out in accordance with relevant guidelines and regulations. All methods are also reported in accordance with ARRIVE guidelines.

### Glucose tolerance and insulin tolerance experiments

In glucose tolerance tests, mice were given 2 g/kg glucose via oral gavage^18^, and blood glucose was measured at 0, 15, 30, 60, and 120 min. For insulin tolerance tests, mice were injected intraperitoneally with 0.5 U/kg insulin, and blood glucose was measured at 0, 15, 30, 45, and 60 min. Prior to the tests, animals experienced a 6-h fasting period and a bedding change.

### Flow cytometry

Spleens were homogenized to obtain single-cell suspensions using a 70 μm nylon strainer. The filtrate was collected, centrifuged to remove the supernatant, and treated with red blood cell lysate for erythrocyte lysis in darkness for 10 min. The resulting cell pellet was resuspended in 1640 medium. For in vitro modeling, Jurkat T cells were cultured in RPMI-1640 complete medium with either normal glucose (5.5 mmol/L) or high glucose (20 mmol/L)^19^. These cells were then seeded into 96-well plates at a density of 10^7^ cells/well and stimulated with 1 μL PMA/ionomycin (MultiSciences, 70-CS1001#) and BFA/monensin (MultiSciences, 70-CS1002#) mixture for 10 h. After stimulation, cells were collected, labeled with CD4-FITC antibodies (Biolegend, anti-human 300,506# and anti-mouse 100,509#) and IL-17-APC antibodies (Biolegend, anti-human 385,903# and anti-mouse 506,915#), and analyzed using flow cytometry.

### Measurement of cytokines

ELISA kits were employed to quantitate the concentrations of cytokines, including TNF-α, IL-17, IL-6, IL-23, following the protocols provided by the manufacturers.

### CCK-8 assay

According to the manufacturer’s instructions, cell viability was assessed using the cell counting kit-8. Jurkat cells were seeded in 96-well microplates, and at specified time intervals (0, 12, 24, 36, 48, and 72 h), 10 μL of CCK-8 reagent was added to each well. The absorbance was measured at 450 nm with a microplate reader.

### Immunofluorescence

Frozen sections were thawed at room temperature for 30 min, followed by antigen retrieval and a 10-min immersion in 0.1% Triton X-100 after TBS washing. Blocking with 5% bovine serum albumin occurred for 30 min at 37 °C. Antibodies against TFF3 Alexa Fluor^®^ 488(#sc-398651 AF488) were purchased from Santa Cruz (Dallas, USA). The antibody was incubated overnight at 4 °C.

### Western blotting

Proteins were extracted using lysis buffer with protease and phosphatase inhibitors. After boiling with loading buffer for 10 min, protein separation occurred through SDS-PAGE, followed by transfer to PVDF membranes. After blocking with 5% nonfat milk for 1.5 h at room temperature, membranes were incubated with primary antibodies overnight at 4 °C. Antibodies against RORγ (#ab113434, 1:1000), IL-17A(#ab193955, 1:1000), and TFF3(#ab272927, 1:1000) were obtained from Abcam (Cambridge, USA). Antibodies against β-actin (#3700S, 1:1000) and GAPDH(#5174S, 1:1000) were obtained from Cell Signaling Technology™ (CST, Danvers, MA, USA). Then, HRP-conjugated secondary antibodies (CAT: 7074# and 7076#, 1:5000, CST, USA) were applied for 1 h at room temperature. Protein bands were detected and quantified using chemiluminescence and Image Lab software.

### Statistical analysis

The data is presented as Mean ± SEM. Statistical analyses were conducted using one-way analysis of variance (ANOVA) along with Dunnett’s T3 multiple comparison test to assess significance among multiple groups. A *p*-value of less than 0.05 was considered statistically significant.

## Results

### T2DM caused splenomegaly and abnormal expression of TFF3 in the spleen

**Figure 1:**
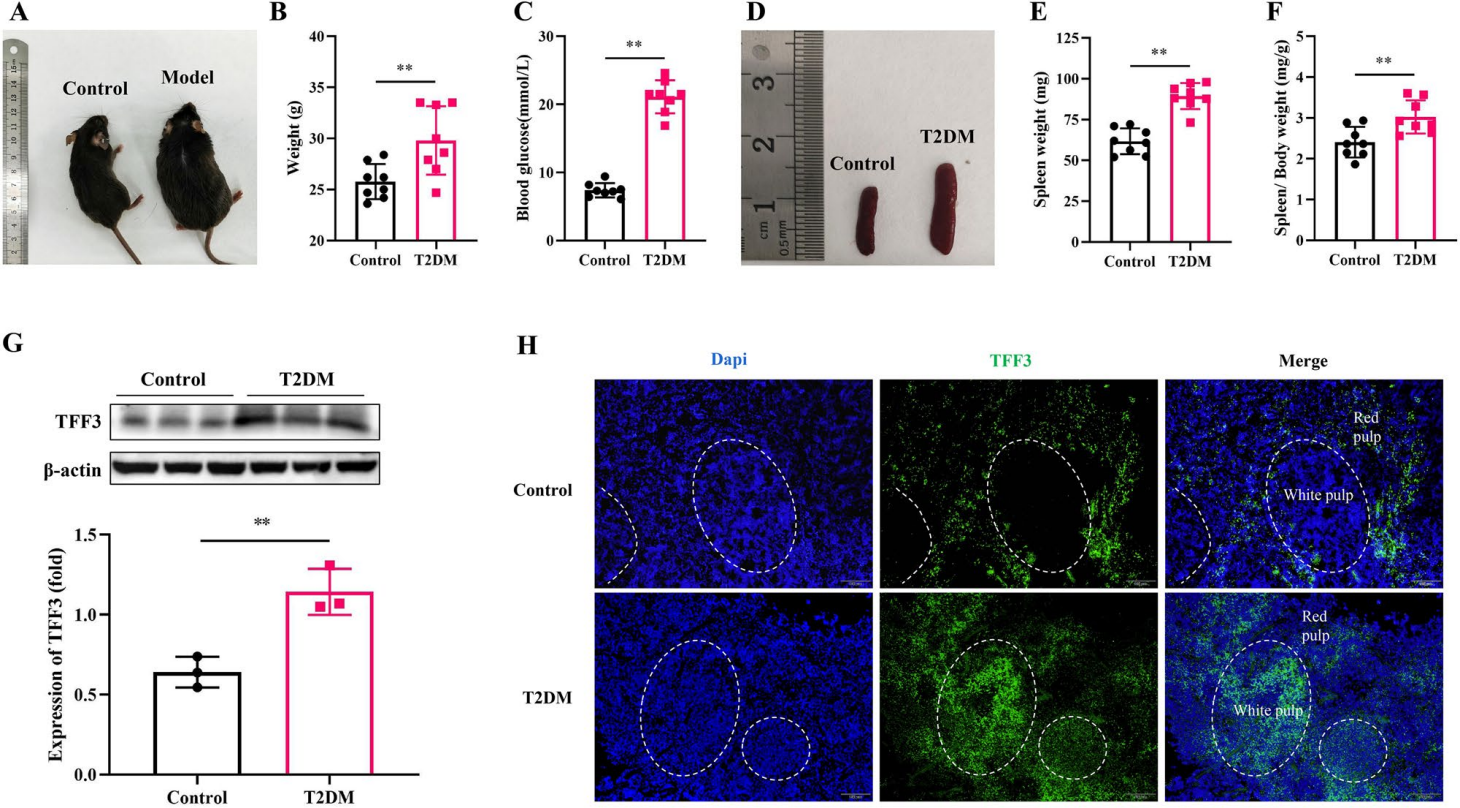
STZ combined with high-fat diet induced obesity, elevated blood glucose, splenomegaly and overexpression of TFF3 in spleen. (**A**) General signs. (**B**) Body weight. (**C**) Blood glucose. (**D**) Spleen morphology. (**E**) Spleen weight. (**F**) Spleen index. The above indicators have n = 8 for each group. (**G**) Proteins levels of TFF3 normalized to β-actin in spleen tissues by western blot, n = 3 in each group. Original blots are included in a supplementary Figure S1. (**H**) TFF3 immunofluorescence in spleen sections from Control (upper panel) and T2DM (lower panel) mice, scale bar = 100 μm. Sections were co-stained for nucleus (blue) and TFF3 (green) as indicated. n = 3 in each group. Values are mean ± SEM. **P < 0.01 versus Control.

Upon general observation, mice in the control group exhibited soft and glossy fur, while those in the T2DM group had dull and oily fur, as depicted in Fig. 1A. In comparison to the control group, the T2DM group displayed significantly increased waist circumference, body weight (*P* < 0.01), and blood glucose levels (*P* < 0.01), indicating the successful establishment of the T2DM model (Fig. 1B,C).

The spleen index, reflective of lymphocyte proliferation and immune function, was notably elevated in mice exposed to STZ in combination with a high-fat diet (Fig. 1D–F), in line with the findings of Xiang et al.^20^. Western blot results revealed minimal TFF3 expression in the spleen of the control group, whereas significant upregulation was observed in the T2DM group (Fig. 1G). Immunofluorescence findings indicated that in the control group, TFF3 was predominantly distributed in the red pulp area of the spleen, with no positive signal detected in the white pulp (Fig. 1H). Conversely, the T2DM group exhibited expression not only in the red pulp but also a gathering of TFF3 in the white pulp. Given that the spleen’s white pulp comprises dense populations of lymphocytes, the heightened TFF3 expression in this region raises the possibility of its interaction with T cells. To explore this hypothesis, we conducted a TFF3 overexpression study on Jurkat T cells in our subsequent investigations.

### TFF3 promoted the differentiation, proliferation and anti-apoptosis of Jurkat T cells cultured in high glucose

**Figure 2:**
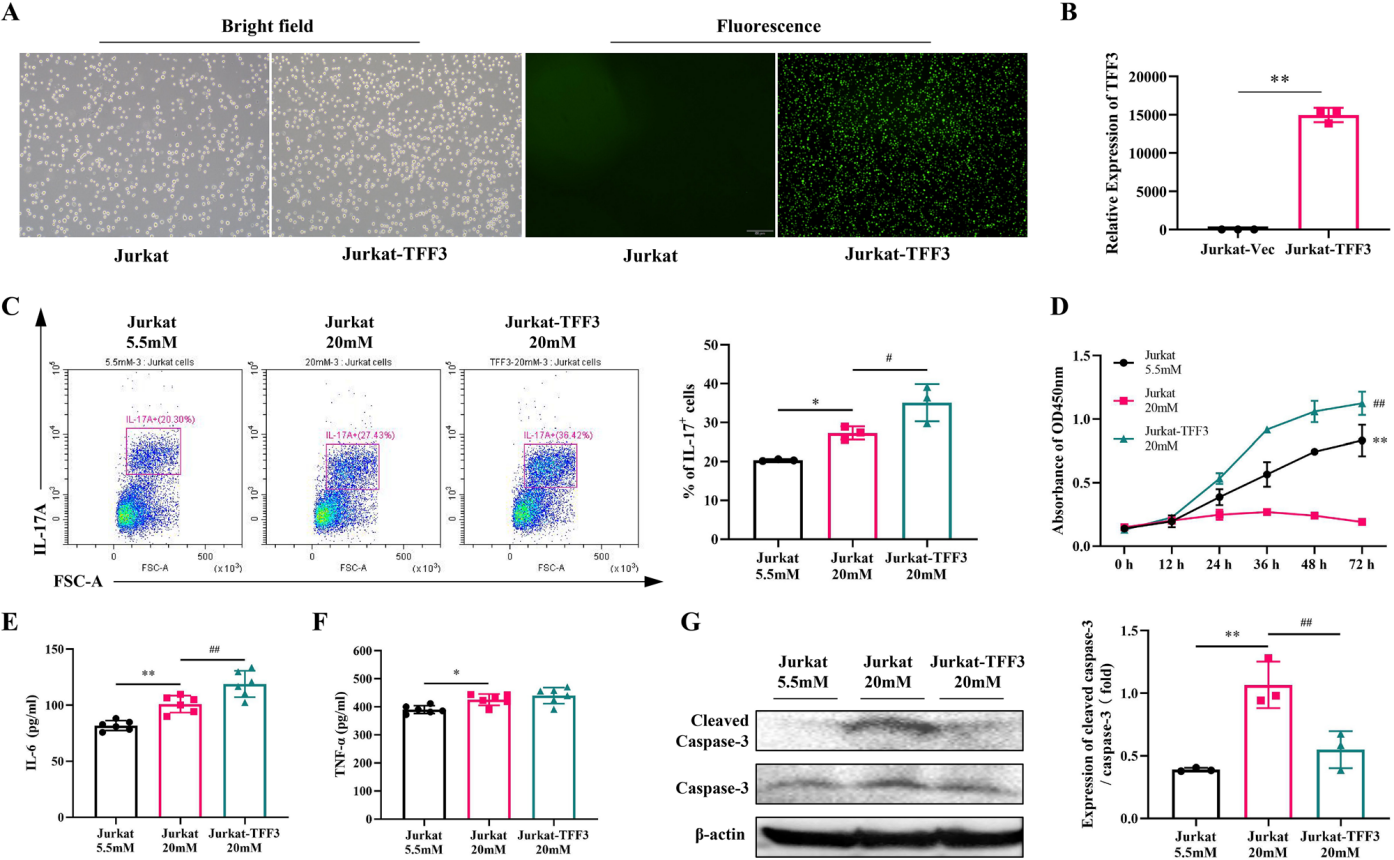
Lentiviral-constructed TFF3 overexpression affected the function of Jurkat cells cultured in high glucose. (**A**) Cell morphology under bright-field and fluorescence microscopy. (**B**) TFF3 gene expression was detected by qPCR. n = 3 in each group. Cells transfected with empty vector (Jurkat-Vec) were used as negative control. (**C**) Analysis of IL-17^+^ (Th17) cells in the Jurkat T cells from normal glucose (5.5 mM) group and high glucose (20 mM) group using flow cytometry (n = 3). Bar charts of the percentage of IL-17^+^ cell. (**D**) Effects of TFF3 on Jurkat T cell proliferation. Cell viability at different time points (0, 12, 24, 36, 48, 72 h) was assessed using the cell counting kit-8 (n = 3). (**E**,**F**) Measurement of TNF-α and IL-6 levels in the medium by ELISA (n = 6). (**G**) Western blot analysis of cleaved caspase-3/caspase-3 in Jurkat cells after treatment with high glucose, normalized with β-actin. n = 3 in each group. Original blots are included in a supplementary Figure S2. Values are mean ± SEM. *P < 0.05, **P < 0.01 vs Jurkat-5.5 mM; ^#^*P* < 0.05, ^##^*P* < 0.01 versus Jurkat-5.5 mM.

We employed a stable lentiviral TFF3 overexpression approach to further support our research. The transfected cells can be seen under a fluorescence microscope to carry green fluorescence (Fig. 2A). The stability of overexpression was confirmed by Real-time PCR (Fig. 2B). Subsequently, the cells were divided into three groups: 1) Jurkat cells cultured in normal glucose (Jurkat-5.5mM); 2) Jurkat cells cultured in high glucose (Jurkat-20mM); 3) Jurkat cells overexpressing TFF3 cultured in high glucose (Jurkat-TFF3-20mM). To observe the differentiation phenotype, we stimulated Jurkat cells with a mixture of PMA/Ionomycin and BFA/Monensin. Ten hours later, the proportion of IL-17A^+^ cells was measured using flow cytometry. The results showed that, compared to the Jurakt-5.5 mM group, there was a significant increase in the proportion of IL-17A^+^ cells in the Jurkat-20 mM group cultured in high glucose (*P* < 0.01), as illustrated in Fig. 2C. Notably, the Jurkat-TFF3-20 mM group exhibited a more substantial increase in the proportion of IL-17A^+^ cells compared to the Jurkat-20 mM group (*P* < 0.05). Furthermore, ELISA results for Th17-related cytokines in the culture medium revealed that the Jurkat-TFF3-20 mM group had higher levels of IL-6 (*P* < 0.05) compared to the Jurkat-20 mM group (Fig. 2E).

CCK-8 assay results demonstrated a significant inhibition of proliferation in Jurkat T cells following long-term high glucose culture compared to the Jurkat-5.5mM group (*P* < 0.01), as shown in Fig. 2D. The proliferation of cells in the Jurkat-20mM group showed a downward trend after 36 h, gradually returning to baseline levels by 72 h. Therefore, we extracted Jurkat T cells cultured for 96 h to detect apoptosis-related proteins. Western blot (WB) analysis revealed that TFF3 overexpression reduced the expression levels of cleaved caspase-3/caspase-3 induced by 96 h of high glucose culture compared to the Jurkat-20mM group (*P* < 0.01), as shown in Fig. 2G. It is worth noting that the viability of the three groups of cells cultured for 12 h showed no difference, remaining at approximately the same level (see Fig. 2D). To observe changes in the phenotype of specific cytokine secretion, it was necessary to ensure that the viability of cells in all three groups remained at a comparable baseline. Therefore, in the experiments shown in Fig. 2C,E, and F, a culture time of 10 h was chosen. Thus, our results demonstrated that TFF3 overexpression enhances the inflammatory phenotype induced by high glucose, which is not dependent on stimulating Jurkat cell proliferation.

### TFF3 knockout improved glucose tolerance in T2DM mice

**Figure 3:**
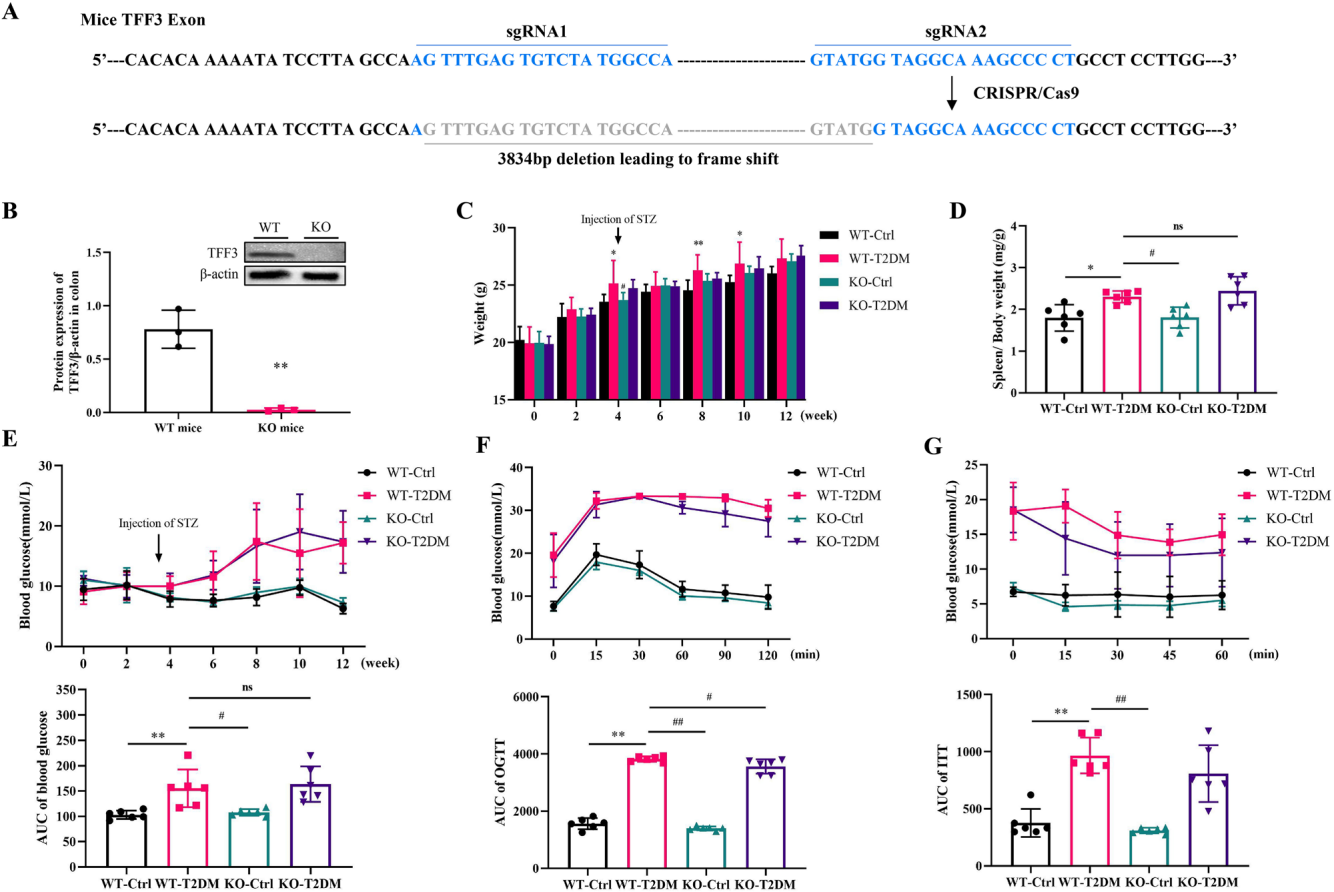
Effects of TFF3 on body weight, blood glucose, spleen index, glucose tolerance and insulin tolerance. (**A**) Schematic illustration of the CRISPR/Cas9 targeting strategy used for generating TFF3 KO mice. The binding sites for guide RNAs are highlighted with boxes. (**B**) Western blot analysis of TFF3 in the colon, with normalization against β-actin (n = 3). Original blots are included in a supplementary Figure S3. (**C**) Body weight changes. (**D**) Spleen index. (**E**) Changes in blood glucose and its AUC. (**F**) Glucose tolerance and its AUC. (**G**) Insulin tolerance and its AUC. The Figures C to G have n = 6 for each group. STZ, streptozotocin; AUC, area under curve; WT, wild tpye; KO, knockout. *P < 0.05, **P < 0.01 versus WT-Ctrl; ^#^*P* < 0.05, ^##^*P* < 0.01 versus WT-T2DM.

We used CRISPR/Cas9 genome-editing technology to generate TFF3^KO^ mice (Fig. 3A,B), followed by the induction of a T2DM model through the combination of STZ injection and a prolonged high-fat diet. Compared to the WT-Ctrl group, the WT-T2DM group exhibited a significant increase in body weight, with notable differences observed during the 4th, 8th, and 10th weeks of the high-fat diet (*P* < 0.05). Conversely, the KO-T2DM group displayed a significant difference during the 12th week of the high-fat diet (*P* < 0.05). However, the weight of the KO-T2DM group was comparable to that of the WT-T2DM group, as depicted in Fig. 3C. Additionally, the weight of the KO-Ctrl group gradually exceeded that of the WT-Ctrl group starting from the second week of the high-fat diet, although no significant differences were observed.

Random blood glucose levels were measured biweekly to construct the blood glucose curve, as illustrated in the Fig. 3E. The results revealed that the area under the curve (AUC) of blood glucose in the T2DM group was significantly higher than that in the WT-Ctrl group. Nonetheless, no notable differences were found between the KO-T2DM group and the WT-T2DM group. Organ index findings also indicated no significant distinctions in spleen index between the KO-T2DM group and the WT-T2DM group (Fig. 3D).

The outcomes of glucose and insulin tolerance tests demonstrated that the AUC in the T2DM group was significantly greater than that in the Control group (Fig. 3F,  *P* < 0.05). This suggests that the combination of STZ and a high-fat diet induced reduced glucose utilization and substantial insulin resistance in mice. Upon comparing the AUC between the WT-Ctrl group and the KO-Ctrl group, as well as between the WT-T2DM group and the KO-T2DM group, we observed that TFF3 knockout mice exhibited a significant improvement in glucose utilization (*P* < 0.05, Fig. 3F) rather than insulin sensitivity (*P* = 0.2316, Fig. 3G) in the glucose and insulin tolerance tests.

### TFF3 knockout suppresses differentiation of splenic T cells into Th17 subtype

**Figure 4:**
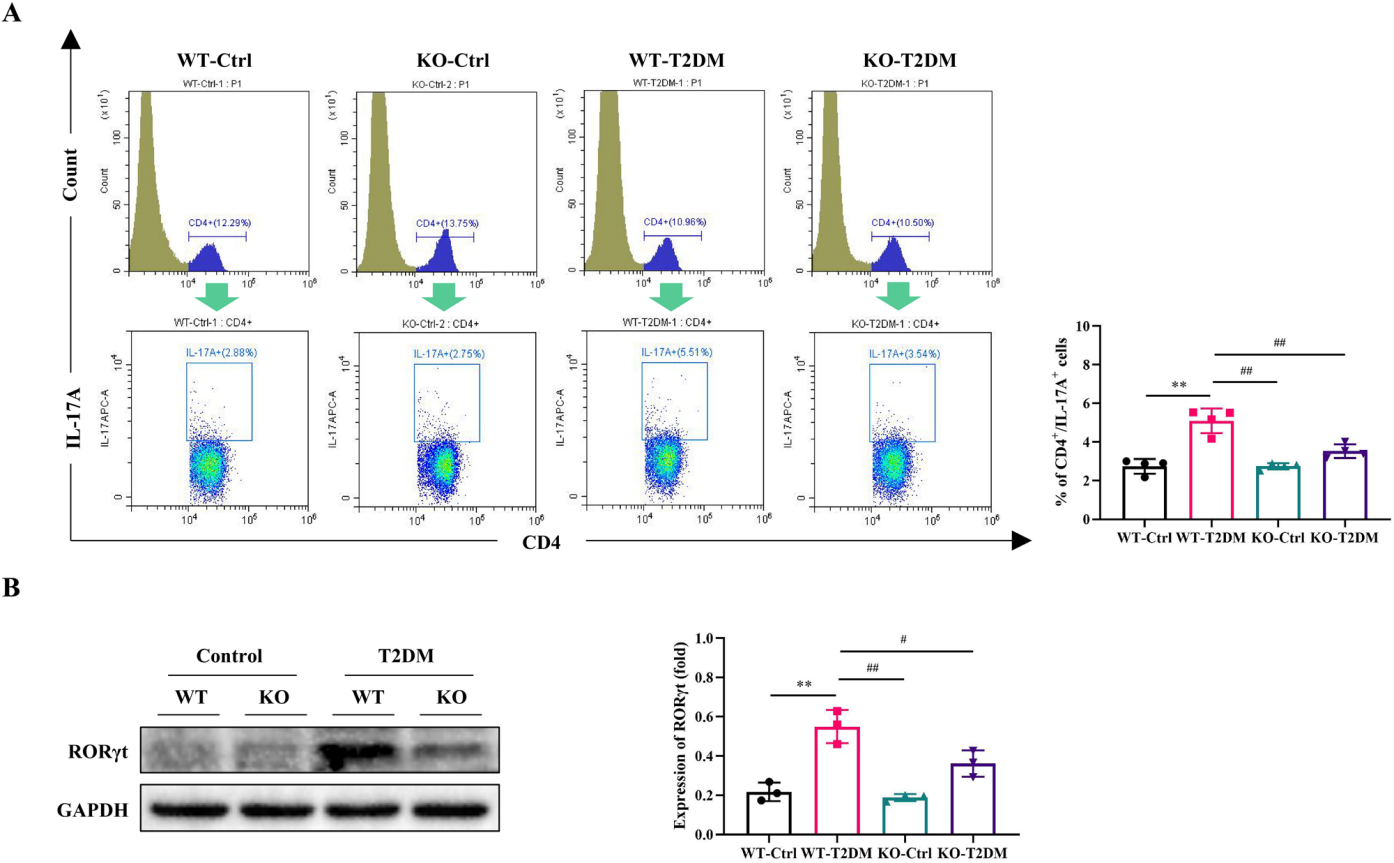
Effects of TFF3 on differentiation of spleen Th17 cells. (**A**) Analysis of CD4^+^IL-17A^+^ (Th17) cells in the spleen using flow cytometry, with bar charts representing the percentage of CD4^+^IL-17A^+^ cells (n = 4). (**B**) Western blot analysis of RORγt in the mouse spleen, with normalization against GAPDH (n = 3). Original blots are included in a supplementary Figure S4. *P < 0.05, **P < 0.01 versus WT-Ctrl; ^#^*P* < 0.05, ^##^*P* < 0.01 versus WT-T2DM.

As the spleen plays a pivotal role in immune responses, we assessed the proportion of inflammatory Th17 cells in the spleen using flow cytometry. Illustrated in Fig. 4A, the population of CD4^+^ IL-17A^+^ cells in WT-T2DM mice was significantly higher than that in the WT-Ctrl group (*P* < 0.01). This observation suggests that the combination of STZ and a high-fat diet induced the differentiation of effector CD4^+^ T cells into Th17 cells. In comparison to the WT-T2DM group, the CD4^+^ IL-17A^+^ cell count in the spleen of the KO-T2DM group exhibited a significant decrease (*P* < 0.05). This finding was further corroborated by Western blot (WB) results, which revealed that the expression of the key transcription factor RORγt in Th17 cells was lower in the KO-T2DM group than in the WT-T2DM group (Fig. 4B).

### Improved inflammation levels and pancreatic injury in TFF3 knockout T2DM mouse

**Figure 5:**
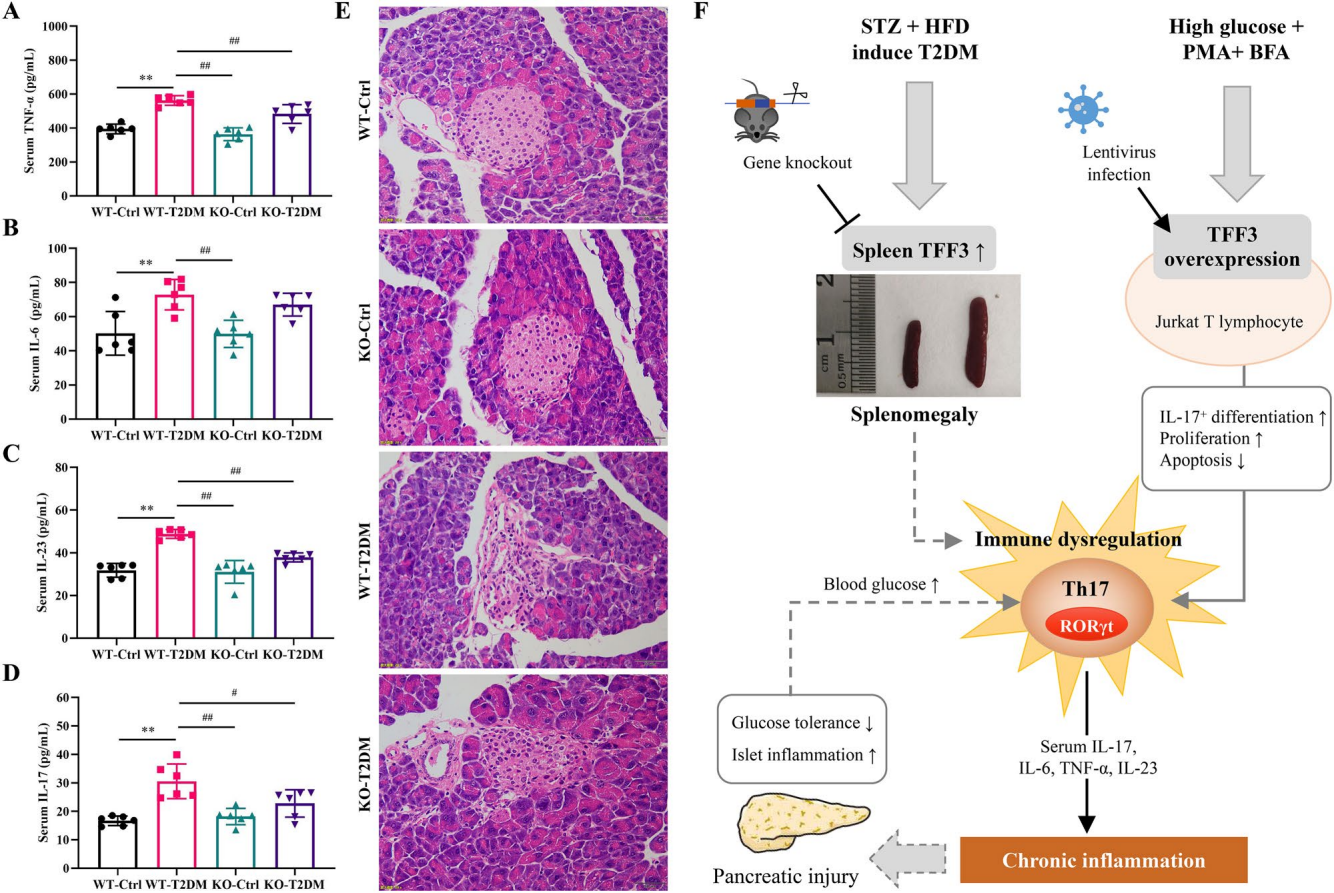
TFF3 knockout improved serum inflammatory levels and pancreatic pathological changes. (**A**–**D**) Levels of TNF-α, IL-6, IL-23 and IL-17 cytokines in the serum were measured by ELISA. n = 6 in each group. (**E**) HE staining of pancreatic tissue (n = 3, 50 μm). *P < 0.05, **P < 0.01 versus WT-Ctrl; ^#^*P* < 0.05, ^##^*P* < 0.01 versus WT-T2DM. (**F**) Schematic diagram of TFF3 involvement in the chronic inflammatory immune response in T2DM. Its mechanism may be related to the regulation of the RORγT/IL-17 signaling pathway and its impact on T cell proliferation and apoptosis. Abbreviation: STZ, streptozotocin; HFD, high-fat diet; T2DM, type 2 diabetes mellitus; WT, wild-type; KO, knockout; Ctrl, control; BFA, Brefeldin A; PMA, phorbol-12-myristate-13-acetate.

Chronic inflammation is intricately associated with diabetes and its related complications. As depicted in Fig. 5A–D, the prolonged exposure to a high-fat diet induced an inflammatory response in WT-T2DM mice, characterized by a significant elevation in serum levels of factors related to Th17 cells, including TNF-α, IL-6, IL-17, and IL-23 (*P* < 0.01, *P* < 0.05, *P* < 0.01, and *P* < 0.01, respectively). In comparison to the WT-T2DM group, the KO-T2DM group exhibited markedly lower levels of TNF-α, IL-17, and IL-23 (*P* < 0.05, *P* < 0.05, and *P* < 0.01, respectively). This suggests that the TFF3 gene knockout reduces the chronic inflammatory response incited by diabetes.

The pancreas is one of the target organs for inflammatory damage. As delineated in Fig. 5E, pancreatic cells in the WT-Ctrl and KO-Ctrl groups displayed an orderly arrangement, with abundant cytoplasm and intact islet cell structures. Conversely, in the WT-T2DM group, pancreatic islet cells exhibited vacuoles, substantial structural alterations, concomitant inflammatory infiltration, and telangiectasia. In contrast to the WT-T2DM group, the islet cells in the KO-T2DM group demonstrated slight deformations, with an overall improvement in the arrangement of pancreatic tissue cells, a reduction in inflammatory infiltration, and amelioration of islet cell vacuolation.

## Discussion

In this study, we present novel findings revealing that the combination of STZ administration with a prolonged high-fat diet induces heightened TFF3 expression within the spleen. Notably, this upregulation is prominent within the white pulp region, which serves as a reservoir for T lymphocytes. Moreover, our study demonstrates that Jurkat cells, an immortalized human T lymphocyte line, exhibit an enhanced proinflammatory phenotype following TFF3 overexpression. This observed phenomenon may be intricately linked to the preservation of Jurkat T cell vitality and their resilience against apoptotic processes. In line with the in vitro findings, the T2DM model established using TFF3^KO^ mice exhibited reduced circulating inflammatory factors and decreased pro-inflammatory Th17 cells in the spleen, along with decreased expression of the key transcription factor RORγt.

Long-term exposure of leukocytes to a high-glucose environment is believed to be a contributing factor to diabetes and its associated complications. These complications are characterized by the induction of inflammatory leukocyte activation and their infiltration into various tissues^21,22^. Activated leukocytes release cytokines that can contribute to tissue inflammation and the formation and maintenance of atherosclerotic plaques. Among leukocytes, lymphocytes are particularly relevant, and exposure to high glucose levels has been shown to induce oxidative stress and increase the expression of Th17 cytokines, including IL-6, IL-17A, and IL-17F^19^. This suggests that hyperglycemia is a risk factor for the activation of lymphocyte Th17 immune responses in diabetes. Our study revealed that following high-glucose, ionomycin, and monensin culture of Jurkat T lymphocytes, a notably increased differentiation of pro-inflammatory Th17 cells was observed in the TFF3 overexpression group. TFF3 not only influenced the differentiation of Jurkat T cells but, as indicated by the results of the CCK-8 assay and Western blotting, also significantly promoted the proliferation and anti-apoptotic capabilities of Jurkat T cells after exposure to high glucose. These effects may contribute to the infiltration of immune cells into organs such as the kidney, liver, and pancreas in diabetes. This phenomenon is consistent with earlier findings, which demonstrated that exposure to TFF2 and TFF3 stimulates the migration of human monocytes^15^. Furthermore, TFF3 has the capacity to interact with chemokine receptors, triggering cell migration through the ERK1/2 and p38 MAPK pathways^23^, which may be associated with immune cell recruitment.

The follow-up effect of chronic inflammation regulated by TFF3 on the target organ is the focus of future work. This article has selected pancreatic tissue for preliminary verification. Our results showed that the pathological changes of pancreas and glucose tolerance of diabetic mice with TFF3 deletion were improved. However, there was no significant difference in body weight, random blood glucose observation for up to 12 weeks, and insulin tolerance test between WT-T2DM group and KO-T2DM group. Therefore, the reduction in the chronic inflammatory response due to TFF3 knockout might not be contingent on effects related to body weight and blood glucose levels. The comprehensive review of TFF3 summarizes the multi-faceted biological functions of TFF3^24,25^. It emphasizes that TFF3 is involved in rather complex regulation in different organs and diseases. In the kidney, Zhang et al. point out that TFF3 protects the kidney from damage of high-sugar microenvironment through enterokidney crosstalk^26^. In the gastrointestinal tract, TFF3 drives anti-helminth immunity by controlling the balance between TH1/TH2 subsets^27^. Under the hypertension model, TFF3 knockout mice with high-salt diet also show suppressed inflammatory response, leading to the decrease of CD25+CD4+T cells in lymph nodes^14^. In the liver, TFF3 deficiency can prevent liver fat accumulation after long-term high-fat diet^28^.

Taken together, TFF3 may play a role in the immune response during the development of T2DM as a regulatory molecule of T cells (Fig. 5F). Its mechanism could involve interference with the RORγt/IL-17A signaling pathway and the regulation of T lymphocyte proliferation and survival. In the future, TFF3 knockout animals may prove to be valuable models for studying metabolic inflammatory diseases, offering significant implications for disease therapy and the development of drugs targeting TFF3.

### Supplementary Information

Supplementary Figures.

## Data Availability

All data generated or analysed during this study are included in this published article [and its supplementary information files].
