# Supplementary figures and images for: Trefoil factor 3 can stimulate Th17 cell response in the development of type 2 diabetes mellitus
Source: Sci Rep. 2024 May 6;14:10340. doi: 10.1038/s41598-024-60426-7 (PMC11074263; doi:10.1038/s41598-024-60426-7)

Figure 1

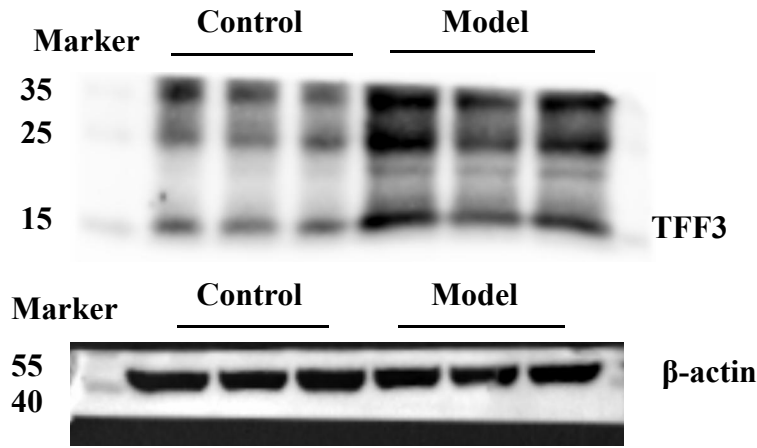

Figure 2

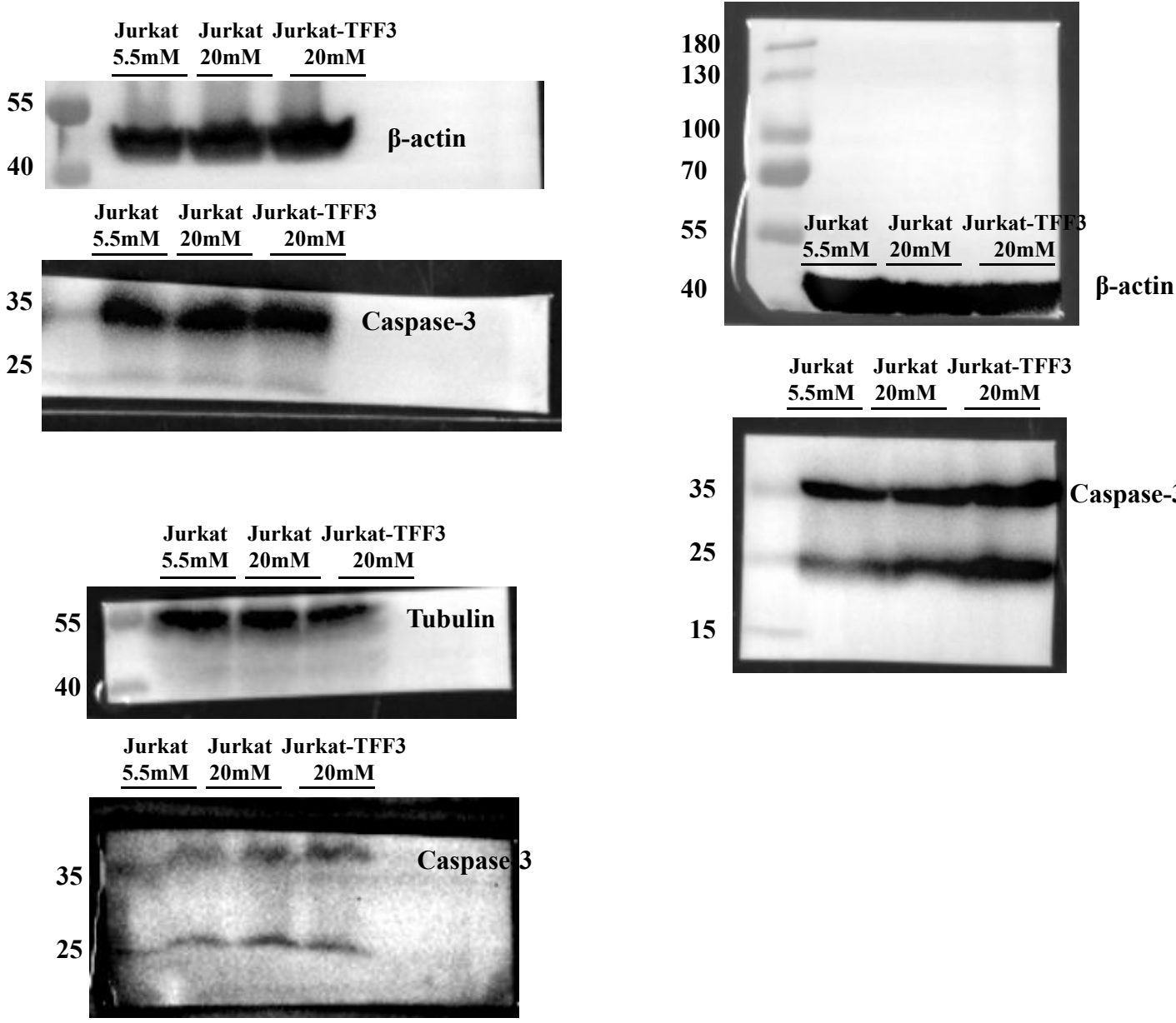

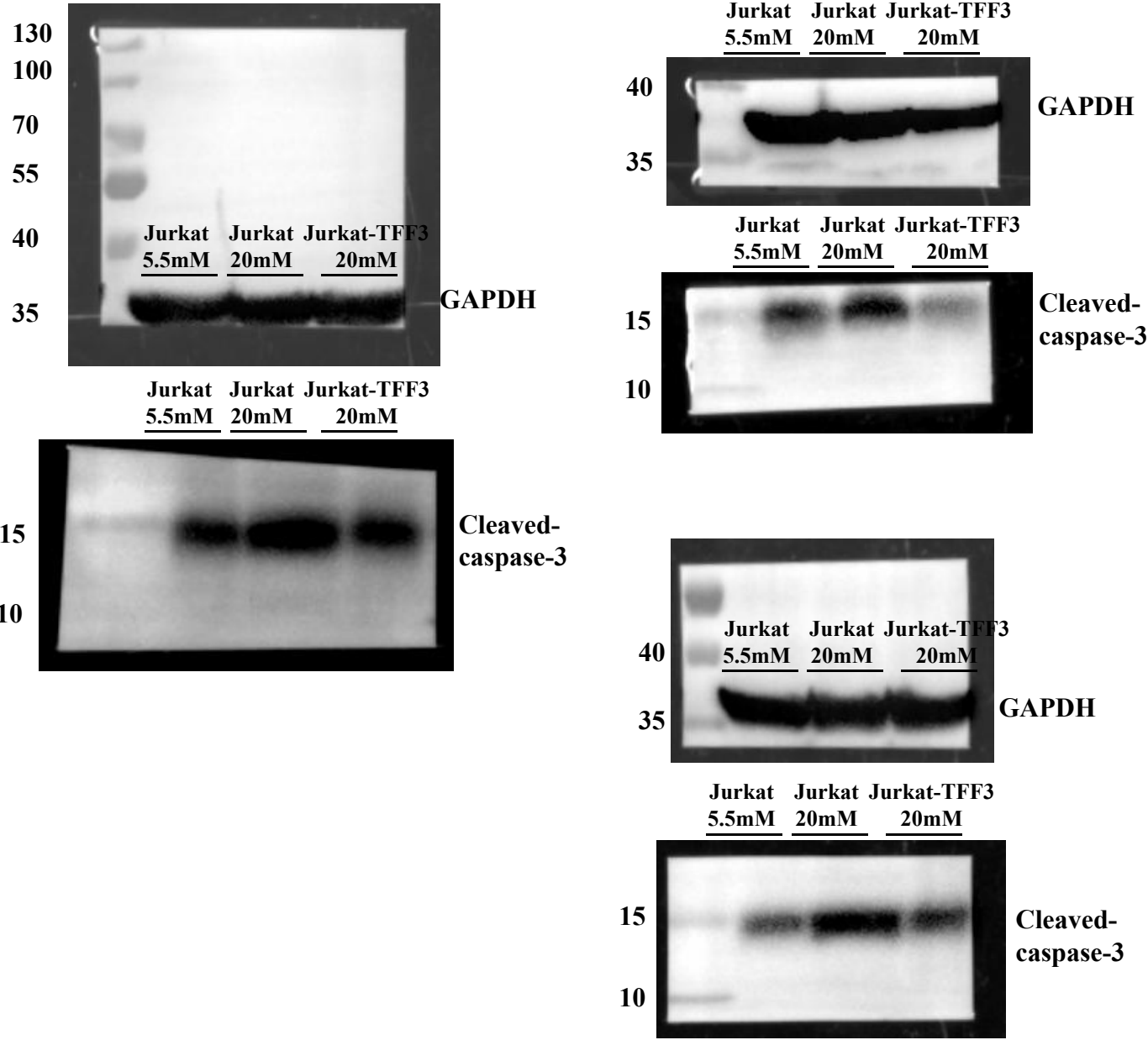

**Figure 3**

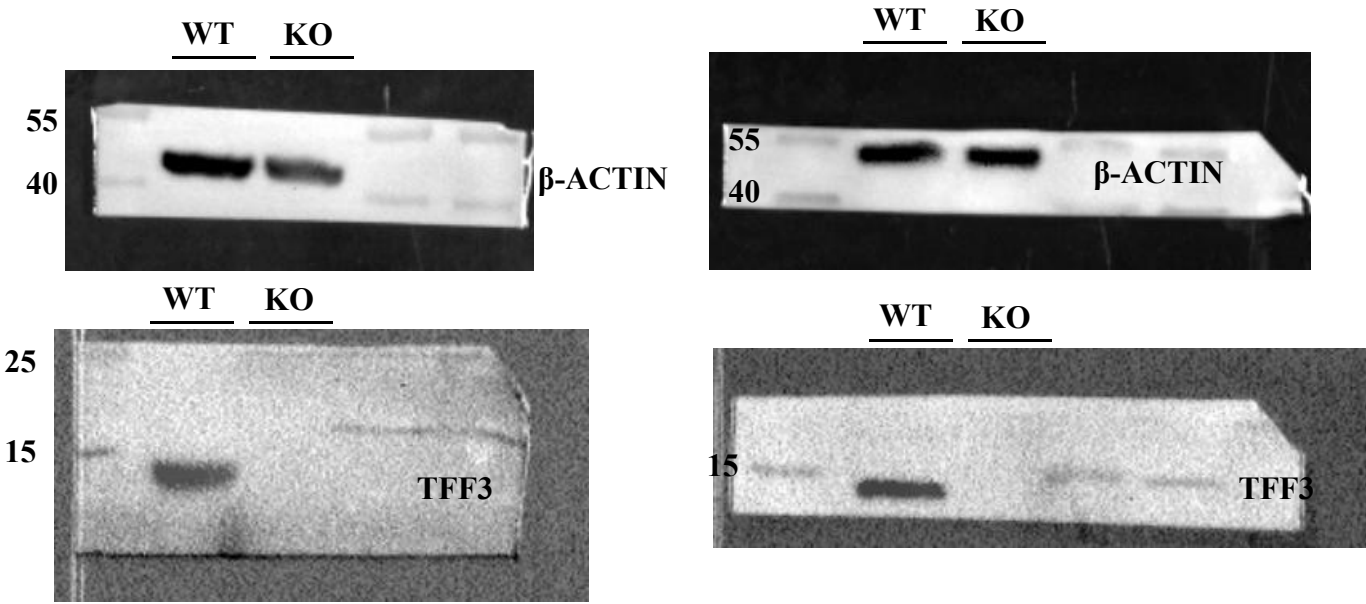

Figure 4

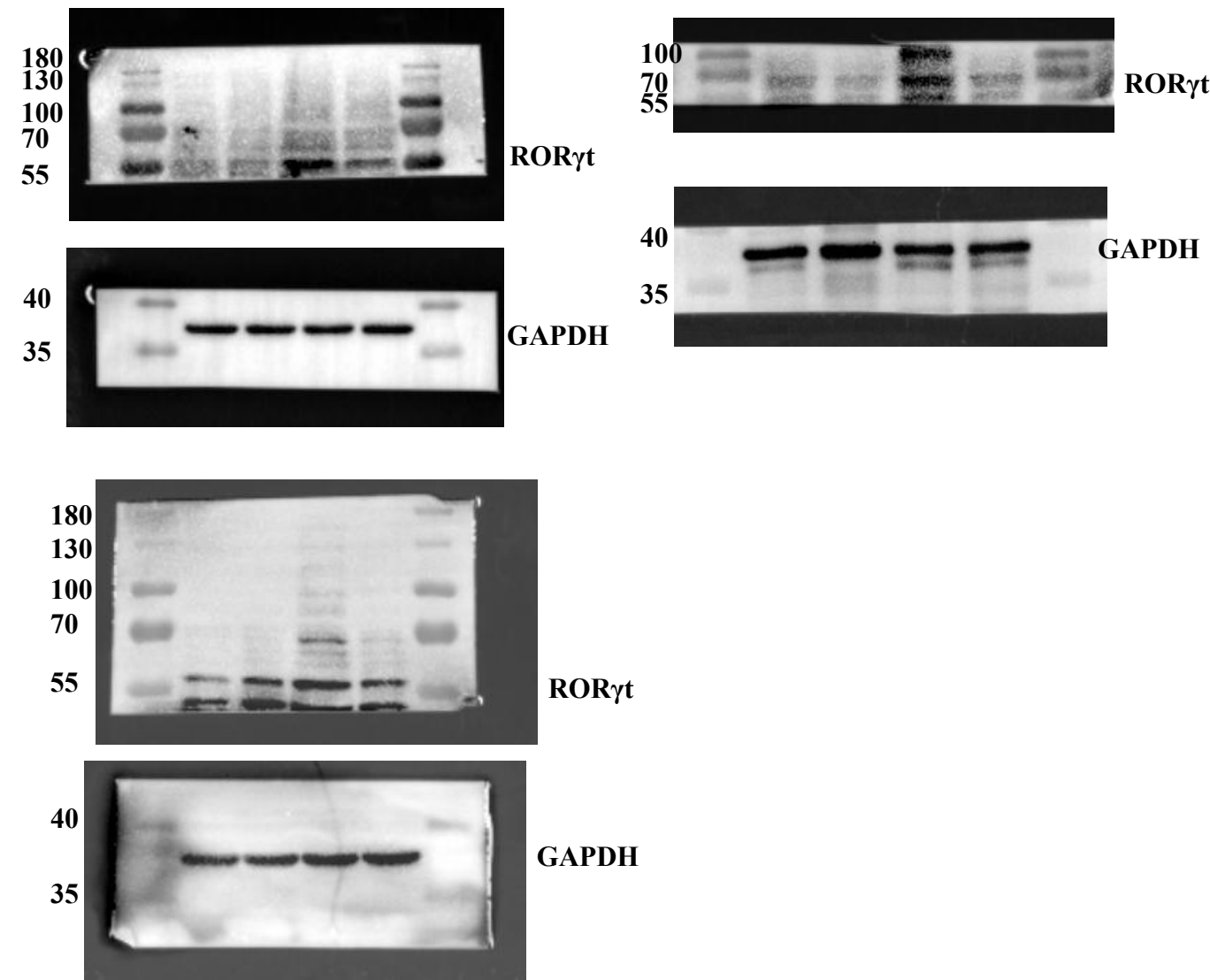

Supplement: Supplementary file 1 — Supplementary Figures. [file 41598_2024_60426_MOESM1_ESM.pdf]
